# Mutational spectrum of *NF1* gene in 24 unrelated Egyptian families with neurofibromatosis type 1

**DOI:** 10.1002/mgg3.1631

**Published:** 2021-06-03

**Authors:** Nahla N. Abdel‐Aziz, Ghada Y. El‐Kamah, Rabab A. Khairat, Hanan R. Mohamed, Yehia Z. Gad, Akmal M. El‐Ghor, Khalda S. Amr

**Affiliations:** ^1^ Medical Molecular Genetics Department Human Genetics and Genome Research Division National Research Centre Cairo Egypt; ^2^ Clinical Genetics Department Human Genetics and Genome Research Division National Research Centre Cairo Egypt; ^3^ Zoology Department Faculty of Science Cairo University Cairo Egypt

**Keywords:** cDNA sequencing, genotype phenotype correlation, MLPA analysis, neuroectodermal disorder, neurofibromin

## Abstract

**Background:**

Neurofibromatosis 1 (NF1; OMIM# 162200) is a common autosomal dominant genetic disease [incidence: ~1:3500]. In 95% of cases, clinical diagnosis of the disease is based on the presence of at least two of the seven National Institute of Health diagnostic criteria. The molecular pathology underlying this disorder entails mutation in the *NF1* gene. The aim of this study was to investigate clinical and molecular characteristics of a cohort of Egyptian NF1 patients.

**Method:**

This study included 35 clinically diagnosed NF1 patients descending from 25 unrelated families. Patients had ≥2 NIH diagnostic criteria. Examination of *NF1* gene was done through direct cDNA sequencing of multiple overlapping fragments. This was supplemented by *NF1* multiple ligation dependent probe amplification (MLPA) analysis of leucocytic DNA.

**Results:**

The clinical presentations encompassed, café‐au‐lait spots in 100% of probands, freckling (52%), neurofibromas (20%), Lisch nodules of the iris (12%), optic pathway glioma (8%), typical skeletal disorders (20%), and positive family history (32%).

Mutations could be detected in 24 families (96%). Eight mutations (33%) were novel.

**Conclusion:**

This study illustrates the underlying molecular pathology among Egyptian NF1 patients for the first time. It also reports on 8 novel mutation expanding pathogenic mutational spectra in the *NF1* gene.

## Graphical abstract

This is the first genetic study of NF1 in North Africa which Investigating NF type I in Egyptian Population, through evaluating NF1 patients frequenting the Genodermatoses Clinic over the past three years for better understanding the disease and its effect on patients’ clinical manifestations. The current study included 35 clinically diagnosed NF1 patients descending from 25 unrelated families. Patients had >2 NIH diagnostic criteria. Examination of NF1 gene was done through direct cDNA sequencing of multiple overlapping fragments. This was supplemented by NF1 multiple ligation dependent probe amplification (MLPA) analysis of leucocytic DNA. The clinical presentations encompassed, café‐au‐lait spots, freckling, neurofibromas, Lisch nodules of the iris, optic pathway glioma, typical skeletal disorders and positive family history. Mutations could be detected in 24 families (96%). Nine of the detected mutations (38%) were novel.

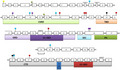

## 1 INTRODUCTION

Neurofibromatosis (NF) is a progressive genetic disease characterized by a neuroectodermal abnormality, mainly affecting the skin, nervous system, bones, eyes, and possibly other organs. This disorder has been divided into three forms: Neurofibromatosis type 1, Neurofibromatosis type 2, and Shwannomatosis (Jett & Friedman, 2010).

Neurofibromatosis 1 (NF1; OMIM# 162200) is one of the most common autosomal dominant genetic diseases with a worldwide incidence of about 1:3500 (Jett & Friedman, 2010; Mao et al., 2018). In 95% of cases, clinical diagnosis of the disease is based on the presence of at least two of the seven National Institute of Health (NIH) diagnostic criteria (“Neurofibromatosis. Conference statement. National Institutes of Health Consensus Development Conference”, 1988) including six or more cafe´‐au‐lait macules (CALs), two or more neurofibromas of any type or one plexiform neurofibroma, inguinal or axillary freckling, optic glioma, two or more Lisch nodules (iris hamartomas), a distinctive osseous lesion such as sphenoid dysplasia or tibial pseudarthrosis and a first‐degree relative with NF1 as defined by the above criteria (Jett & Friedman, 2010). In some cases, the disease has also been manifested with learning disabilities, vascular disease, skeletal abnormalities, central nervous system (CNS) neoplasms, or malignant peripheral nerve sheath tumors (MPNSTs; Jett & Friedman, 2010). Before the age of 8 years, many cases do not meet a sufficient number of classical diagnostic criteria and cannot be diagnosed clinically (Sabatini et al., 2015). Although NF1 is a classical monogenic disorder with complete penetrance by adulthood, clinical symptoms can vary within a family, or even at different life stages of the same patient (Mao et al., 2018). The reasons for phenotypic variability are poorly understood, but it could be due to modifier genes, epigenetic alterations, or other environmental factors (Sabatini et al., 2015).

The NF1 disease is caused by heterozygous mutations in the neurofibromin gene (17q11.2; NM_001042492.2; Barker et al., 1987). It is a tumor suppressor gene whose protein, neurofibromin down regulates Ras‐GTP levels in the Ras/MAPK/AP‐1 pathway (Brundage et al., 2014). The NF1 protein contains six main domains. The Ras‐GAP activity of NF1 protein is mediated by the GTPase activating protein Related Domain (GRD), which corresponds to exons 27–34 (Abramowicz & Gos, 2014). The *NF1* (OMIM# 613113) gene is expressed in many human tissues, such as brain, white blood cells, skin fibroblast, spleen, muscle, and lung. Expression of NF1 was also reported in tumor tissues, such as neuroblastoma, neurofibroma, thymoma, and breast cancer. Suzuki et al. reported the expression of NF1 in NF1 neurofibrosarcoma cell line, and a colon carcinoma cell line (Suzuki et al., 1991). The alternatively spliced isoforms of the NF1 transcript have been investigated to determine their expression pattern in various tissues (Shen et al., 1996).

The *NF1* is one of the longest protein coding genes in the human genome and contains 61 exons distributed over 350 Kb (Trovo‐Marqui & Tajara, 2006). The gene codes for at least four alternatively spliced transcripts (Shilyansky et al., 2010). The two major protein isoforms are type I (GRD I) and type II (GRD II; 2818 and 2839 amino acids, respectively; Trovo‐Marqui & Tajara, 2006). They are tissue specific where neurofibromin type I predominates in neurons of CNS and dorsal root ganglia, while type II predominates in most other tissues and in Schwann cells, and is essential for learning and memory in mouse models (Barron & Lou, 2012). It was shown that GRD I and GRD II are equally represented in Epstein–Barr virus transformed lymphocytes either from NF1 patients or from normal controls and also, in human placenta, kidney, and lung (Suzuki et al., 1991; Viskochil et al., 1993). Andersen et al showed that GRD I and GRD II transcripts were expressed in various tissues but with a variation in their relative amounts (Andersen et al., 1993). It was found also that the expression of the two isoforms was associated with the differentiation status of a particular tissue. GRD I predominated in the fetal brain and undifferentiated primitive neuroectodermal tumors, whereas GRD II was predominantly expressed in differentiated cell lines.

The molecular study of *NF1* genes is a challenging process because of the *NF1* gene size and its several alternatively spliced transcripts. The gene has the highest mutation rate seen in humans (estimated at 1 in about 10,000 alleles per generation); approximately 100‐folds higher than those seen for other loci (Shen et al., 1996), and about 50% of the cases are caused by sporadic mutations (Valero et al., 2011; Peltonen & Pöyhönen, 2012). Mutations are dispersed throughout the *NF1* gene with no identified hot‐spot mutation and there is a wide spectrum of the different types of mutations (Peltonen & Pöyhönen, 2012). Moreover, multiple *NF1* pseudogenes are found in the human genome and some of these pseudogenes are expressed, thus additionally complicating specific primer design.

More than 2,500 different *NF1* mutations have been reported and listed in the Human Gene Mutation Database Professional (Stenso et al., 2003). Most of the mutations (93%) are small mutations (including nonsense, missense, insertion, deletion or splicing mutations). The remaining ones consist of intragenic deletions/duplications (2%) and microdeletions that span NF1 and neighboring genes (5%). These mutations could be identified mainly by multiplex ligation‐dependent probe amplification (MLPA) analysis (Terribas et al., 2013). Therefore, it might be more efficient and economical to primarily use cDNA sequencing rather than DNA sequencing for *NF1* sequence analysis.

Although genotype–phenotype correlations have been proposed, further investigations are required to confirm their validity. There are several reported genotype–phenotype correlations. First, individuals with large (~1.4 Mb) genomic microdeletions, spanning the entire *NF1* gene locus and neighboring genes, have more severe clinical phenotype, including increased number of neurofibromas, elevated risk for cardiac malfunction, skeletal anomalies, facial dysmorphism, malignant tumor development, and a higher prevalence of learning disabilities compared to patients with an intragenic *NF1* mutation (Pasmant et al., 2010). Second, a specific germline *NF1* gene mutation (c.2970_2972delAAT) do not cause the development of cutaneous neurofibromas in NF1 patients (Upadhyaya et al., 2007). The third genotype‐phenotype correlation was the association between the different changes of the amino acid located at p. Arg1809 and a milder form of the disease. This form is characterized by the presence of CALs and freckles only (Pinna et al., 2015; Rojnueangnit et al., 2015; Santoro et al., 2015). Recently a new genotype‐phenotype correlation was reported by Koczkowska et al. in which the pathogenic *NF1* p. Met1149, p. Arg1276, or p. Lys1423 missense variants had an association with a Noonan‐like phenotype (Koczkowska et al., 2020). In this correlation, p. Arg1276 and p. Lys1423 pathogenic missense variants were associated with a high prevalence of cardiovascular abnormalities, while p. Arg1276 variants had a high prevalence of symptomatic spinal neurofibromas compared with “classic” NF1‐affected cohorts. However, p. Met1149‐positive individuals had a milder phenotype, characterized mainly by pigmentary manifestations without presence of neurofibromas.

To the best of our knowledge, this is the first report investigating the molecular pathology of NF1 among Egyptian cases. Here, we report on 35 Egyptian patients to elucidate the mutational spectrum among them and investigate possibilities of genotype‐phenotype correlation.

## 2 SUBJECTS AND METHODS

### 2.1 Subjects

The present study included 35 patients ascertained from 25 unrelated families that were originated from different Egyptian governorates. Patients were referred to the Genodermatoses Clinic, National Research Centre (NRC) Cairo, Egypt. Diagnosis of NF1 disease was based on the presence of two or more features of the NIH diagnostic criteria (“Neurofibromatosis. Conference statement. National Institutes of Health Consensus Development Conference”, 1988).

All patients and sibs were subjected to complete clinical examination and full medical history including three generation‐pedigree construction. Clinical evaluation also comprised brain neuroimaging (CT and/or MRI), electroencephalogram (EEG), karyotyping, fundus examination, and echocardiography.

### 2.2 Methods

A written consent was obtained from all participants in accordance with the ethical standards of the institutional and/or national researchethical committee (Medical Research Ethics Committee at the National Research Center; Reference Number: 15‐221) and with the 1964 Helsinki declaration and its later amendments or comparable ethical standards.

### 2.3 DNA and RNA isolation

Peripheral blood samples of all patients and their parents were obtained.

Total RNA extraction from peripheral blood leucocytes was done using QIAamp RNA Blood Mini Kit (Qiagen, Hilden, Germany) according to the manufacturer's protocol. To prevent illegitimate splicing, blood samples were processed within 4 hours from venipuncture. Genomic DNA was extracted from peripheral blood using a standard method (Miller et al., 1988) for performing MLPA analysis and verification of the novel mutations detected in cDNA samples.

### 2.4 NF1 mutation analysis by cDNA sequencing approach

Reverse transcription was performed using 500 ng of total RNA and random hexamers according to the manufacturer's instructions of High‐Capacity cDNA Reverse Transcription Kit (Thermo Fisher Scientific Inc.). The entire coding region of the *NF1* gene (GenBank Ref Seq no. NG_009018.1) was amplified in 16 overlapping fragments. 25‐uL final reaction mix containing 2.0 uL of cDNA, 10 pmol each primer, 200 mmol/L dNTPs, and 1X reaction buffer with 1.5 mmol/L MgCl_2_ and 1.5 U GoTaq® G2 Flexi DNA Polymerase (Promega). Amplification conditions were as follows: 95°C for 5 min, followed by 35 cycles of 95°C for 1 min, annealing temperature (range: 57°C–66°C) for 1 min, and 72°C for 1 min for 35 cycles. The final extension was 72°C for 10 min. Primer pairs were designed from the reference sequence of the *NF1* gene (GenBank Ref Seq no. NG_009018.1, NM_001042492.2; Ensembl transcript ID ENST00000358273.8) [Designed primer sequences are available on request]. Quality of primers was examined using NetPrimer software and the product was blasted by NCBI nucleotide blast software. Subsequently, The PCR products were purified using Exo‐SAP PCR Clean‐up kit (Fermentas) and sequenced in both directions using the BigDye Terminator v3.1 Cycle Sequencing Kit (Applied Biosystems). Furthermore, verification of the mutations detected by cDNA analysis was carried out by sequencing of the genomic DNA regions encompassing them using BigDye Sequencing Kit. Detected mutations were described according to isoform type II (NCBI accession no. NM_000267.3; Ensembl transcript ID ENST00000356175.7). Variants were annotated in accordance to HGVS nomenclature.

### 2.5 MLPA analysis

For patients with no detected pathogenic mutations by sequencing, their samples were further analyzed using SALSA MLPA P081/P082 NF1 Kit for single and multiple exon deletions/duplications, according to the manufacturer's instructions (MRC Holland). Peak areas for each separated fragment were measured using the Coffalyser.NET software (Version v. 140721.1958, MRC Holland).

### 2.6 Database search and bioinformatics analyses

All the genetic variants identified were queried by browsing through different databases including the LOVD (Leiden Open Variation Database [Fokkema et al., 2011]), Human Gene Mutation Database (HGMD; “HGMD® home page”, 2003) and NCBI dbSNP (database of Single Nucleotide Polymorphisms, ClinVar). Reported frequencies of identified variants were verified on 1000 genomes (“IGSR | samples”, Clarke 2017) and the gnomAD (“NF^1^ | gnomAD”, 2019) databases.

For novel missense mutations, the putative effects on the NF1 protein were investigated using several prediction algorithms and scoring tools, including SIFT (Sim et al., 2012), Polyphen2 (Adzhubei et al., 2010), MutPred (Xie et al., 2013), Mutation Assessor (Reva et al., 2007), REVEL (Ioannidis et al., 2016) SNP&GO (Calabrese et al., 2009), PhD‐SNP (Capriotti et al., 2006), PROVEAN (Choi et al., 2012), and Mutation Taster (Schwarz et al., 2014).

## 3 RESULTS

### 3.1 Clinical findings

This study included 35 clinically diagnosed NF1 patients who were ascertained from 25 families, probands’ clinical features are listed in Table1. Positive family history was noted in 32% of patients (8/25) and the rest were sporadic. For familial cases, four was paternally inherited and four was maternally inherited. Parental consanguinity was reported in 36% of families (9/25). The six NIH diagnostic criteria were present in probands with different percentages: 1—CALs (≥6 Patches), 100% (25/25); 2—Freckling, 60% (15/25); 3—neurofibromas, 20% (5/25); 4—Lisch nodules of the iris, 12% (3/25); 5—Magnetic Resonance Image (MRI) evidence of optic nerve glioma, 8% (2/25); and 6—distinctive osseous lesions, 20% (5/25). Minor features were present in 80% (20/25) of probands (Table 1). Karyotyping was normal for all Probands.

**TABLE 1:** Clinical criteria of the enrolled patients

Patient ID	Age (at diagnosis, years)	Consanguinity	Gender	≥ 6 cafe au lait macules	Freckling	≥ 2 neurofibromas	Optic glioma	≥ 2 Lisch nodules	A distinct osseous lesion	First degree relative with NF1	Minor clinical features
F1/III‐1	18	Positive	Female	+	−	+	−	−	−	−^a^	MRI showed UBOs
F2/VI‐1	9 months	Positive	Female	+	+	−	−	−	Scoliosis	−	Hypotonia, mild valvular pulmonary stenosis and dysmorphic features.
F3/III‐2	4	Negative	Female	+	+	−	−	−	deformed back/neck (lateral deviation)	−	MRI showed bilateral basal ganglia, cerebellar, and brain stem multiple patches; gingival racial pigmentation
F4/V‐2	3	Negative	Male	+	−	−	−	+	−	−	Scalp hemangioma
F5/III‐3	7 months	Negative	Male	+	+	−	−	−	−	−	Dysmorphic face and asymmetry
F6/III‐2	4	Negative	Male	+	+	−	−	−	−	−	Delayed speech, thick corpus callosum, hypoplastic vermis, and increased BG signal
F7/III‐1	6	Negative	Male	+	+	−	−	−	−	−	Congenital cataract
F8/VI‐1	2	Positive	Male	+	+	−	−	−	Genu valgum	+	macrocephaly, right hearing loss, and delayed speech
F9/III‐1	9 months	Negative	Female	+	+	−	−	−	−	−	MRI showed right internal capsule ischemic insult
F10/V‐2	9 months	Positive	Male	+	−	−	−	−	−	+	Hypotonia and hyporeflexia
F11/IV‐3	1	Positive	Male	+	+	−	−	−	−	−	Dysmorphic Features
F12/III‐1	1	Negative	Male	+	−	−	+	−	sphenoid wing dysplasia	−	MRI showed optical canal dilation
F13/IV‐3	1	Positive	Male	+	+	+	−	−	−	+	Bilateral simian creases
F14/VI‐11	3	Negative	Male	+	−	−	−	−	Spine affection and deformed chest	−	Delayed developmental milestones and delayed speech
F15/III‐1	31	Negative	Male	+	+	−	−	−	−	−	—
F16/III‐2	13	Positive	Female	+	−	−	−	−	−	+	3 fibromas
F17/V‐6	6	Positive	Female	+	+	−	−	−	−	−	—
F18/III‐1	12	Negative	Male	+	+	−	−	−	−	−	
F19/III‐1	6	Negative	Female	+	+	−	−	−	−	−^a^	Delayed developmental milestones
F20/III‐2	5	Negative	Female	+	+	−	−	−	−	−	Subnormal mentality and poor achievement, dry hands, dysplastic nails, and delayed bone age Short stature
F21/III‐4	3	Negative	Male	+	+	−	−	−	−	+	delayed speech
F22/III‐1	6	Negative	Male	+	_	+	−	−	−	+	—
F23/III‐3	2.5	Negative	Female	+	−	−	−	+	−	+	—
F24/V‐7	13	Positive	Male	+	+	−	−	−	−	−	Mitral valve stenosis, moderate pulmonary valve regurge and moderate pulmonary hypertension, thick corpus callosum
F25/III‐2	9	Negative	Male	+	+	−	+	+	−	+	Subnormal mentality and poor school achievement, MRI showed left ganglionic solitary focus of signal alteration

Abbreviations: −, clinical feature is absent; +, clinical feature is present; BG, basal ganglia; MRI, Magnetic Resonance Image; UBO, unidentified bright objects in brain imaging studies.

^a^ Father unavailable for study.

### 3.2 Molecular findings

Mutational analysis of the entire coding region of the *NF1* gene identified pathogenic mutations in 24/25 families (96%; Table 2). The majority of detected mutations (22/24, 92%) consisted of point mutations and small indels (≤4 bp). They comprised nonsense mutations (6/22; 27%), single nucleotide duplications (3/22; 14%), small deletions (3/22; 14%), missense mutations (7/22; 32%), splice site mutations (2/22; 9%), and inframe deletions (1/22; 4%).

**TABLE 2:** Molecular analysis of NF1 mutations identified in this study

Patient ID	Exon NCBI nomenclature NG_009018.1 (Old nomenclature)	Genomic position^a^	cDNA position NM_000267.3 Ensembl transcript ID ENST00000356175.7	Protein position	Type of mutation	Effect on mRNA level	Status	ClinVar accession number of novel mutations	Reference
F1/III‐1	Exon 1	g.384A>C	c.1A>C	p.?	start loss		Novel	SCV000999060	This study
F2/VI‐1	intron 4–5 (4a−4b) (DNA)	g.74963A>G	c.480‐2A>G	–	Splice Site	deletion of 7 bp p.(Leu161Asnfs*2) c.481_487del	Reported		Kluwe et al. (2002) Ref ID: rs1567820688 HGMD ID: CS020536
F3/III‐2	Exon 12 (10a)	g.111331G>A	c.1278G>A	p.(Trp426*)	Nonsense		Reported		Griffiths et al. (2007) Ref ID: rs1131691085 HGMD ID: CM076346
F4/V‐2	Exon 16 (12a)	g.128552_128555delACTA	c.1756_1759delACTA	p.(Thr586del)	Frameshift		Reported		Klose et al. (1999) Ref ID: rs786202782 HGMD ID: CD982825
F5/III‐3	Exon 18 (13)	g.131548C>T	c.2041C>T	p.(Arg681*)^b^	Nonsense		Reported		Ars et al. (2000) Ref ID: rs768638173 HGMD ID: CM00002
F6/III‐2	Exon 18 (13)	g.131593_131595delTGG	c.2086_2088delTGG	p.(Trp696del)	Inframe deletion		Novel	SCV000999062	This study
F7/III‐1	Intron 19–20 (14–15) (DNA)	g.132366G>T	c.2325+1G>T	–	Splice Site	skipping of Exon 19	Novel	SCV000999063	This study
F8/VI‐1	Exon 21 (16)	g.134135C>T	c.2446C>T	p.(Arg816*)	Nonsense		Reported		Maynard et al. (1997) HGMD ID: CM971040
F9/III‐1	Exon 21 (16)	g.134210A>C	c.2521A>C	p.(Thr841Pro)	Missense		Novel	SCV000999064	This study
F10/V‐2	Exon 23 (18)	g.135341C>T	c.2998C>T	p.(Arg1000Cys)^b^	Missense		Reported		Pasmant et al. (2015) Ref ID: rs367684252 HGMD ID: CM153412
F11/IV‐3	Exon 23 (18)	g.135447T>A	c.3104T>A	p.(Met1035Lys)	Missense		Reported		Giugliano et al. (2019) Ref ID: rs137854553
F12/III‐1	Exon 26 (20)	g.137911dupA	c.3452dupA	p.(Asn1151Lysfs*44)	Frameshift or PTC		Novel	SCV000999065	This study
F13/IV‐3	Exon 27 (21)	g.138158T>G	c.3579T>G	p.(Phe1193Leu)	Missense		Novel	SCV000999066	This study
F14/VI‐11	Exon 28 (22)	g.140697C>T	c.3721C>T	p.(Arg1241*)	Nonsense		Reported		Fahsold et al. (2000) Ref ID: rs137854562 HGMD ID: CM000799
F15/III‐1	Exon 31 (24)	g.163511A>C	c.4267A>C	p.(Lys1423Gln)	missense		Reported		Li et al. (1992) Ref ID: rs137854550 HGMD ID: CM920506
F16/III‐2	Exon 36 (28)	g.2309692delT	c.4911delT	p.(Leu1638Serfs*39)	Frameshift or PTC		Reported		Side et al., (1997), van Minkelen et al. (2014) HGMD ID: CD1415185
F17/V‐6	Exon 36 (28)	g.230976_230979delAAGT	c.4918_4921delAAGT	p.(Lys1640Glyfs*36)	Frameshift or PTC		Novel	SCV000999067	This study
F18/III‐1	Exon 38 (30)		ex. 38. c.5484‐?_5686+?del (Exon38 deletion)		Large deletion		Reported		Imbard et al. (2015) HGMD ID: CG155143
F19/III‐1	Exon 39 (33)	g.239938>T	c.5839>T	p.(Arg1947*)	Nonsense		Reported		Cawthon et al. (1990) Ref ID: rs137854552 HGMD ID: CM900173
F20/III‐2	Exon 45 (37)	g.243742dupG	c.6784dupG	p.(Asp2262Glyfs*24)	Frameshift		Novel	SCV000999068	This study
F21/III‐4	Exon 45(37)	g.243750C>A	c.6791C>A	p.(Tyr2264*)	Nonsense	skipping of Exon 45	Reported		Messiaen et al. (1997) Ref ID: rs772295894 HGMD ID: CM981382
F22/III‐1	Exon 45 (37)	g.243749dupA	c.6791dupA	p.(Tyr2264*)	stop gain		Reported		Upadhyaya et al. (1996) Ref ID: rs876657715 HGMD ID: CI962317
F23/III‐3	Exon 47 (39)	g.248138T>G	c.7118T>G	p.(Leu2373Arg)	Missense		Reported with Uncertain clinical significance		Ref ID: rs1135402899
F24/V‐7		Heterozygous whole gene deletion					Reported		Wu‐Chou et al. (2018)

^a^ Variant annotations are in accordance to HGVS nomenclature.

^b^ gnomAD allele frequency for the variants Arg681Ter & Arg1000Cys is 3.98899E‐06 & 1.2e‐5, respectively; gene accession number: (GenBank Ref Seq no. NG_009018.1; NM_000267.3; Ensembl transcript ID ENST00000356175.7).

**FIGURE 1:**
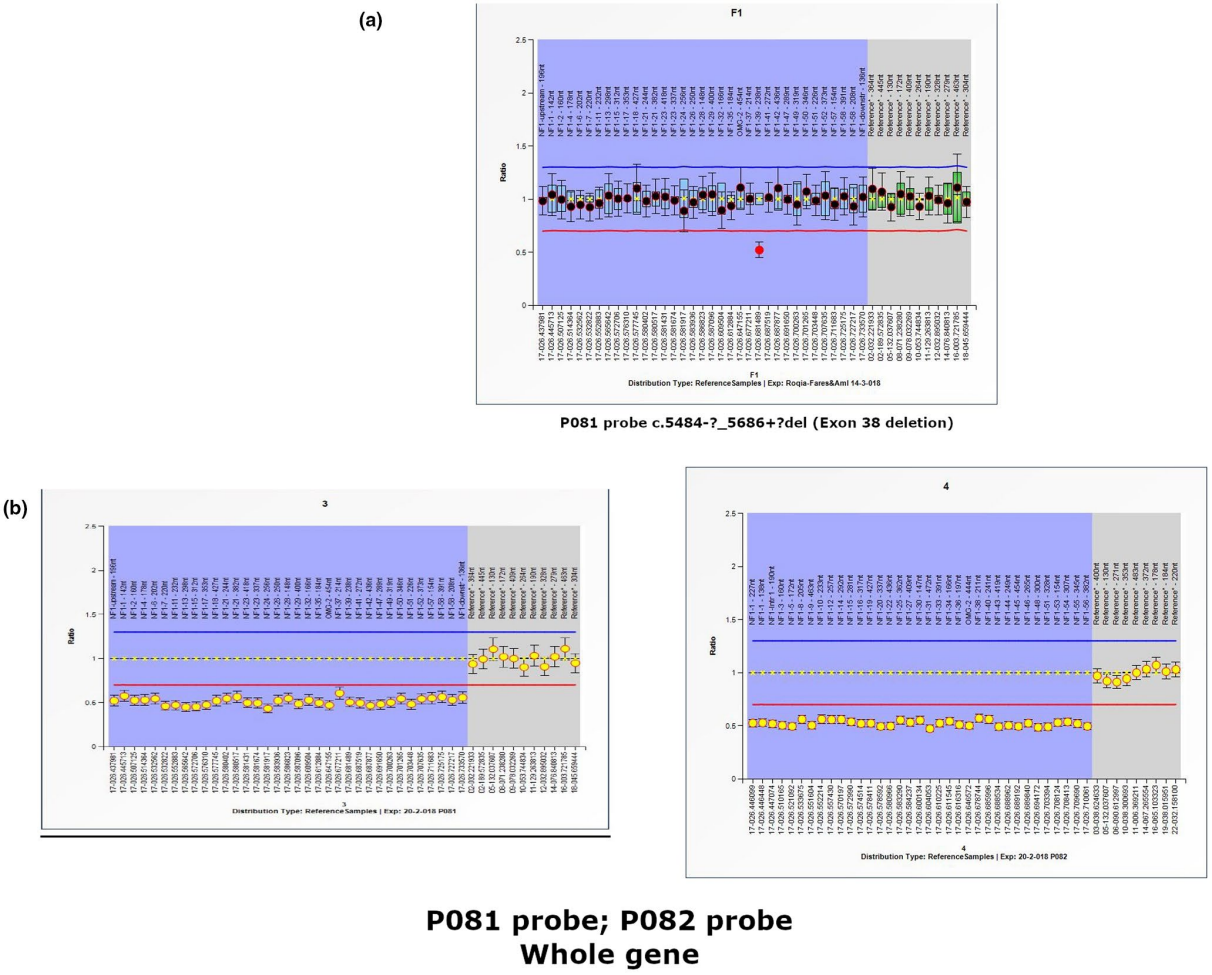
MLPA ratio charts of patients with detected variation. (a) Patient (F18/III‐1), deletion of exon 38 indicated; (b) patient (F24/V‐7), deletion of the whole gene indicated gene accession number: (GenBank Ref Seq no. NG_009018.1; NM_000267.3; Ensembl transcript ID ENST00000356175.7)

In addition, MLPA analysis revealed the presence of gross deletions in 2 patients (8%). Whole *NF1* gene deletion in one patient (F22/V‐7) and one exon deletion in another (F16/III‐1; Figure 1).

**FIGURE 2:**
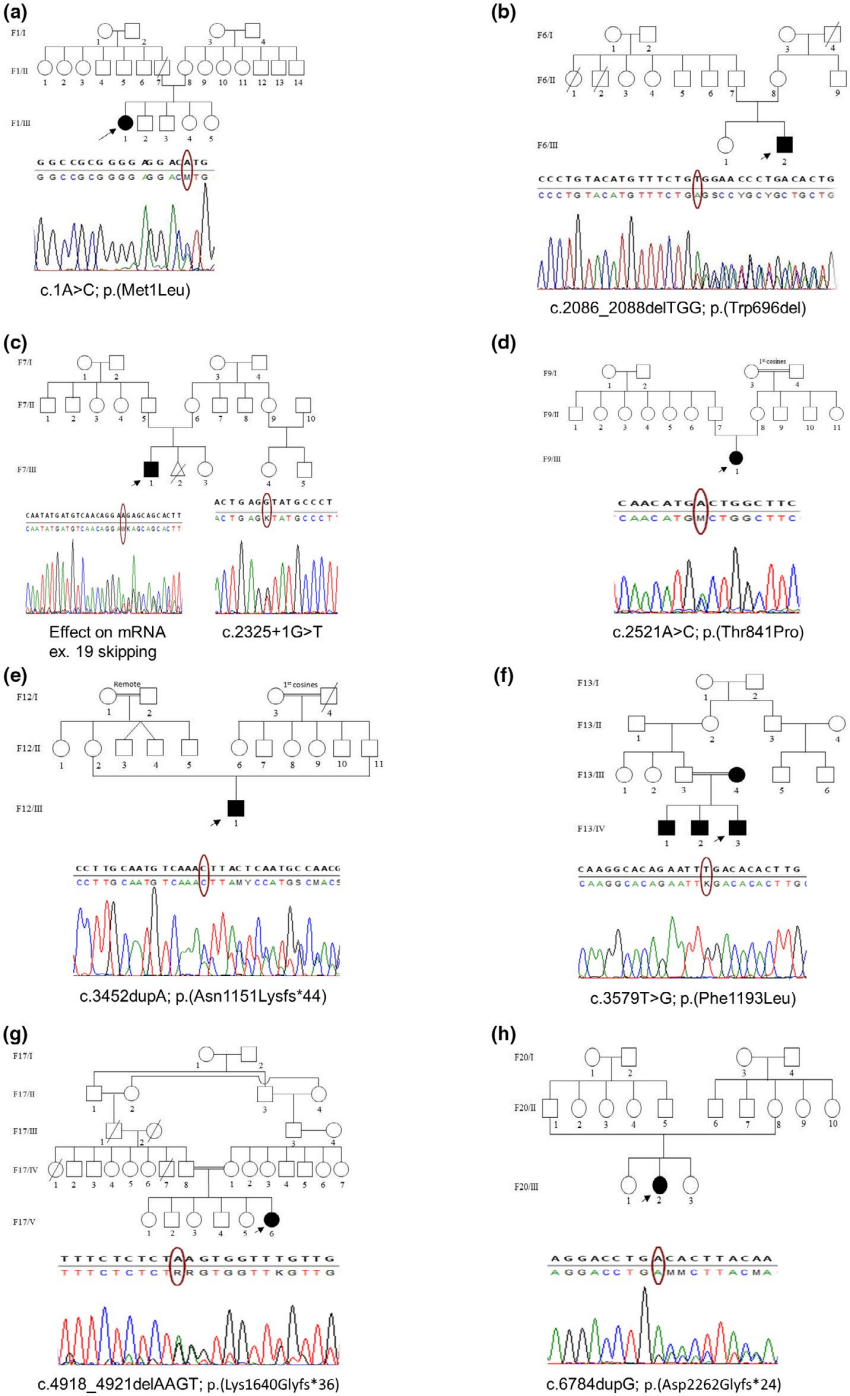
Pedigrees and 8 novel mutations detected by Sanger sequencing in 8 patients. The arrows on the pedigree indicate the probands in each family. Ellipse shape the sequence indicates position of mutation. Mutations: (a) patient (F1/III‐1), c.1A>C; (b) patient (F6/III‐2), c.2086_2088delTGG; (c) patient (F7/III‐1), c. 2325+1G>T; (d) patient (F9/III‐1), c.2521A>C; (e): patient (F12/III‐1), c.3452dupA; (f) patient (F13/IV‐3), c.3579T>G; (g) patient (F17/V‐6), c.4918_4921del AAGT; (h) patient (F20/III‐2), c.6784dupG. gene accession number: (GenBank Ref Seq no. NG_009018.1; NM_000267.3; Ensembl transcript ID ENST00000356175.7). ○, Normal female; □, Normal male; ●, Affected female; ■, Affected male; 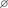, Deceased female; 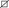, Deceased male; 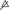, Aborted fetus; 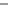, positive consanguinity

A total of eight identified mutations (33%) were novel; 3 of them were frameshift mutations, 1 inframe deletion, 3 missense mutations, and 1 splice mutation, (Figure 2). Only one of these novel mutations was detected in a proband familial case (F13/IV‐3) but the other seven were de novo. Novel mutations have been identified and already submitted to ClinVar database.

### 3.3 In silico analysis

Four out of the eight novel mutations were variants causing an unambiguous pathogenic effect on the NF1 protein (frameshifting, nonsense, and nucleotide changes at the canonical ±1 or 2 splice site; Richards et al., 2015).

The inframe mutation c.2086_2088delTGG (p. Trp696del) was predicted by PROVEAN to have a deleterious effect (score: 16.512).

The effect of the 3 missense variants were predicted with 9 algorithms and scoring tools including: SIFT, Polyphen2, Mutation Assessor, REVEL, Mutation Taster, MutPred, PROVEAN, PhD‐SNP, and SNPs&GO (Table 3). Mutation c.1A>C (p. Met1Leu) was predicted to have a pathogenic effect in 4 algorithms Whilemutation c.2521A>C (p. Thr841Pro) was predicted to be deleterious or has a pathogenic effect in 7 algorithms. The pathogenicity of the third missense mutation c.3579T>G (p. Phe1193Leu) was confirmed in all the nine algorithms. These missense variants were classified either likely pathogenic variants (class 4) or pathogenic variants (class 5) according to ACMG recommendations (Richards et al., 2015).

**TABLE 3:** In silico analysis of Novel missense and uncertain mutations.

Mutation	PhD‐SNP	Mutation assessor	PROVEAN	SNPs&GO	REVEL	SIFT	POLYPHEN2	Mutation taster	MutPred
c.1A>C p.(Met1Leu)	Neutral (RI = 7)	NE	Neutral (−1.561)	Disease (RI = 10)	Likely Benign (0.356)	Tolerated (0.11)	probably damaging (0.924)	Likely Disease causing	Deleterious (0.886) Altered Transmembrane protein (0.9; 0.05) Loss of N‐terminal acetylation at M1 (0.02; 7.0e−03)
c.2521A>C p.(Thr841Pro)	Neutral (RI = 1)	Low (1.865)	Deleterious (−4.738)	Disease (RI = 10)	Likely Disease Causing (0.936)	Damaging (0)	probably damaging (0.996)	Disease causing (38)	Deleterious (0.943)
c.2998C>T p.(Arg1000Cys)	Disease (RI = 2)	Medium (0.842)	Deleterious (−5.53)	Disease (RI = 10)	Likely Disease Causing (0.761)	Damaging (0)	probably damaging (0.981)	Disease causing (180)	Deleterious (0.832) Altered Disordered interface (0.19; 0.04)
c.3579T>G p.(Phe1193Leu)	Disease (RI = 3)	Medium (2.695)	Deleterious (−4.983)	Disease (RI = 10)	Likely Benign (0.316)	Damaging (0)	probably damaging (0.98)	Disease causing (22)	Deleterious (0.654) Altered Transmembrane protein (0.24; 1.6e−03)
c.7118T>G p.(Leu2373Arg)	Disease (RI = 7)	Medium (1.965)	Deleterious (−5.13)	Disease (RI = 10)	Likely Disease Causing (0.708)	Damaging (0.00)	probably damaging (0.993)	Disease causing (102)	Deleterious (0.945) Gain of Allosteric site at H2393 (0.19; 0.05)

Abbreviations: MutationAssessor, predicted functional, i.e. high (“H”) or medium (“M”), or predicted non‐functional, i.e. low (“L”) or neutral (“N”). score cutoffs between “H” and “M”, “M” and “L”, and “L” and “N”, are 3.5, 1.935 and 0.8, respectively; NE, not evaluated; PhD‐SNP &SNPs&GO, directly predicts disease effect and give Reliability Index range from 0 to 10. 0 unreliable & 10 reliable; PROVEAN, scores ≤‐2.5 is predicted as “Damaging”; otherwise it is predicted as “Neutral”; REVEL, PolyPhen2, and MutPred score ranges from 0.0 (tolerated) to 1.0 (deleterious) it also give the predicted effect on the Molecular mechanisms with *p* ≤ 0.05 (probability; *p*‐value); SIFT, threshold for intolerance is 0.05.

## 4 DISCUSSION

Neurofibromatosis type I is one of the most common autosomal dominant diseases. Despite its monogenic nature, it is characterized by an extremely variable clinical presentation even within the same family. Identification of the genetic causes of the NF1 disease has greater diagnostic utility because mutation detection can confirm the etiology of the disease in individuals in whom the clinical phenotype does not fulfill the NIH diagnostic criteria. A firm diagnosis of NF1 directs the patient toward a multidisciplinary management with specific monitoring. It allows counseling regarding mode of inheritance, recurrence risk, and potentially prenatal diagnosis too. However, detection of the pathogenic variations in *NF1* gene is a challenging process due to the large size of the gene, lack of hot spot mutations and presence of several transcripts and pseudogenes. In this study, we integrated MLPA and cDNA analyses to detect the underlying mutations in *NF1* gene in an Egyptian patient cohort. We successfully identified the causative mutation in 24 out of 25 NF1 patients (detection rate: 96%). Up to our knowledge, this is the first molecular report on Egyptian NF1 patients. Generally, there are only few studies about NF1 in the Middle East including two large cohort studies in Tunisia (Azaiz et al., 1994; Gouider et al., 1994), and another one in United Arab Emirates (Ben‐Salem et al., 2014).

The detected mutations in the current study included 7 missense, 6 nonsense, 2 splice site, 6 frameshift, and 1 inframe mutation, in addition to 2 gene and exon deletions. The rate of de novo mutations was 68%, higher than previous reports (~50%; Valero et al., 2011; Peltonen & Pöyhönen, 2012).

The detected mutations were scattered along the whole gene starting from the start codon in which there was a novel mutation (c.1A>C, p. Met1Leu) in patient F1/III‐1. This mutation was predicted to be likely pathogenic according to ACMG recommendations. Other mutations in the start codon were reported before, and postulated that one of the downstream inframe methionine (M68, M102 and M108) might act as an alternative start codon (Fahsold et al., 2000). Another hypothesis postulated that Leu might act as non‐AUG start codon where it could replace AUG for translation initiation (Lind & Aqvist, 2016). However, experimental functional studies are required to reveal the consequences of such mutations.

Two other mutations reside in the non‐characterized region located before the first domain, Cystein/Serine Rich Domain (CSRD). One mutation is a previously reported splice site mutation (c.480‐2A>G) detected in a 9 month old infant (F2/VI‐1) who manifested with CALs, Freckling, and scoliosis (Upadhyaya et al., 2008). The second (c.1278G>A, p. Trp426*), a previously reported nonsense mutation, was detected in the patient F3/III‐2 presenting with CALs, freckling, and deformed back/neck (lateral deviation; Sabbagh et al., 2013). So it might be suggested that a correlation between molecular pathology of this non‐characterized region and skeletal anomalies is possible.

Six mutations were identified (6/24, 25%) in the CSRD (residues 543–909). Clustering of mutations in this domain had previously been observed (Fahsold et al., 2000; Mattocks et al., 2004). It is hypothesized that CSRD domain binds to ATP and has three cAMP‐dependent protein kinase (PKA) recognition sites that are phosphorylated by PKA (Izawa et al., 1996). Mangoura et al. (2006), showed that neurofibromin Ras‐GAP activity is regulated by PKC‐dependent phosphorylation of CSRD and proposed that CSRD phosphorylation may increase Ras‐GAP function and promote the arrest of cell growth instead of cell proliferation. Xu et al. (2018), proposed a positive association between optic nerve glioma (OPG) and NF1 mutation clustering in CSRD. The OPG was not evidenced, so far, in any of our six patients (F4/V‐2, F5/III‐3, F6/III‐2, F7/III‐1, F8/VI‐1, and F9/III‐1) with CSRD mutations. Three of these mutations were novel, including 1 inframe mutation c.2086_2088delTGG (p. Trp696del) detected in patient F6/III‐2. This mutation was predicted by PROVEAN to have a deleterious effect. The second mutation c. 2325+1G>T was a splice site detected in patient F7/III‐1 and predicted to be disease causing by mutation taster prediction software. Importantly, this mutation causes skipping of exon 19 on the mRNA level, which confirms splice site change and its pathogenicity. The third novel mutation in patient F9/III‐1 was a missense mutation (c.2521A>C, p. Thr841Pro) and predicted to be a likely pathogenic variant (class 4) according to ACMG recommendations. Additionally, three previously reported CSRD mutations were identified, one frameshift mutation c.1756_1759delACTA in patient F4/V2 and two nonsense mutations (c.2041C>T; p. Arg681* in patient F5/III‐3 and c.2446C>T; p. Arg816* in F8/VI‐1). This later mutation was reported by several research groups to correlate with different types of neurofibromas and severe clinical manifestations (Fahsold et al., 2000; Upadhyaya et al., 2008).

In between the first domain (CSRD) and the second domain, tubulin binding domain (TBD), there is an unidentified region consisting of 2 exons (23 and 24). In this region, we detected two missense mutations. Both mutations were previously reported, the first one was (c.2998C>T; p. Arg1000Cys in F10/V‐2; Pasmant et al., 2015). Although the mutation was reported in ClinVar with uncertain significance, insilico analysis supported its pathogenicity (Table 3). The second mutation (c.3104T>A; P. Met1035Lys) was recently reported (Giugliano et al., 2019). Our patient F11/IV‐3 shared the NIH criteria with the patient reported by Giugliano et al. (i.e. CALMs and Freckles only). However they were different in the minor features as the patient in the current study presented only dysmorphic features while the previously reported patient presented dystrophic scoliosis, and behavioral problems (Giugliano et al., 2019).

The TBD interacts with the dynein heavy chain and kinesin‐1 ‐ motor proteins responsible for transporting organelles or large complexes along the cytoskeleton fibers (Arun et al., 2013). Interaction of NF1 with motor proteins will therefore be particularly important for the proper functioning of cells having long cytoplasmic protrusions, such as melanocytes or Schwann cells (Abramowicz & Gos, 2014). Two novel mutations were detected in this domain; a frameshift mutation (c.3452dupA, p. Asn1151Lysfs*44 in F12/III‐1) and a missense mutation (c.3579T>G, p. Phe1193Leu) in family 13 that encompassed 4 affected cases.

The activation of Ras catalyzed GTP hydrolysis by Neurofibromin is well investigated in biochemical and structural detail. The importance of Arg1276 for the physiological functionality of Neurofibromin was underlined by demonstration that the missense mutation Arg1276pro led to a 8000‐fold reduction of Neurofibromin GAP activity in vitro, without changing the binding affinity towards Ras (Klose et al., 1998). This provided direct evidence that neurofibromin GAP function failure was the critical element in NF1 pathogenesis. In our cohort, only two NF1 mutations affected GRD‐GTPase domain. Both mutations were previously reported, the first one was a nonsense mutation (c.3721>C>T; p. Arg1241*) in patient F14/VI‐11 with a relatively severe phenotype (Table 1). Due to its occurrence in a hypermutable CpG dinucleotide, this mutation was a recurrent one and might be mediated by 5‐methylcytosine deamination (Krawczak, 1994). The second one was a missense mutation in exon 31(c. 4267A>C; p. Lys1423Gln). It was identified in a 31 year old patient (F15/III‐1), who presented only with CALs and Freckles. His clinical presentation was milder than those of the 2 previously reported patients with the same mutation. The first patient presented with CALs, macrocephaly, hypertelorism and cardiac abnormalities in the form of pulmonary valve stenosis while the second presented with CALs, hypertelorism, Lisch nodules, malar hypoplasia and pectus/thoracic abnormalities in addition to minor features (De Luca et al., 2005).

Two frameshift mutations in exon 36 (c.4911delT; p. Leu1638Serfs*39 in F16/III‐2 and c.4918_4921delAAGT, p. Lys1640Glyfs*36 in F17/V‐6) were detected in the fourth domain of NF1 protein, SEC14p Homology Domain (SEC 14).. The first case was a 13 year teenage girl who developed CALs at age of one year but increased in number and size with age and also had 3 truncal fibromas. The first mutation was previously reported in two NF1 patients from different populations, one of them had malignant myeloid disorder (van Minkelen et al., 2014; Side et al., 1997). The second mutation was novel and the patient presented only with CALs and freckles.

In the third unidentified region between the Pleckstrin Homology (PH), and the C‐terminal Domains (CTD), one repeatedly reported mutation (c.5839C>T; p. Arg1947* in F19/III‐1) in exon 40 was detected (Fahsold et al., 2000; Park et al., 2000). It was suggested that the Arg1947 codon is a mutation hot spot and that this cytosine is particularly prone to mutation (Park et al., 2000). This mutation was reported previously in different patients with different clinical presentations.

Four mutations were detected in the last NF1 domain, CTD. One of them was a novel frameshift mutation (c.6784dupG, p. Asp2262Glyfs*24) in a 5 year old girl (F20/III‐2) with severe phenotype. The other three mutations were previously reported, two of them were two different nonsense mutations in exon 45 (p. Tyr2264*). The first nonsense mutation (c.6791C>A; p. Tyr2264*) was identified in a 3 year child (F21/III‐1) presented with CALs and delayed speech with difficulty in attiring special sounds. It is worth mentioning that Messiaen et al, reported two different patients with the same mutation who had learning disability (Messiaen et al., 1997) which could be a possible genotype phenotype correlation among our patients and previously reported ones. This mutation was also among few mutations detected in several NF1 reports (Ars et al., 2000). Interestingly this mutation led to exon skipping rather than protein truncation as in the most cases of nonsense mutations (Upadhyaya et al., 1996). The second nonsense mutation (c.6791dupA; p. Tyr2264*) which caused protein truncation was detected in family 22 as well as previously reported in patients presenting with CALs, neurofibroma, and/or Scoliosis (Maruoka et al., 2014; Upadhyaya et al., 1996). The third previously reported mutation in this domain was missense mutation (c.7118T>G; p. Leu2373Arg) in patient F23/III‐3. Although the mutation was reported in ClinVar with uncertain significance, according to ACMG guidelines this mutation had two strong evidences, so it is recommended to be pathogenic.

In our cohort, two different large mutations were detected by MLPA analysis. One exon deletion (c.5484‐?_5686+?del) was detected in a sporadic case (F18/III‐1) who presented with CALs and Freckles. The second one was a whole gene deletion in case F24/V‐7 who developed neither learning disabilities nor facial dysmorphism like the previously reported one, (Pasmant et al., 2010).

**FIGURE 3:**
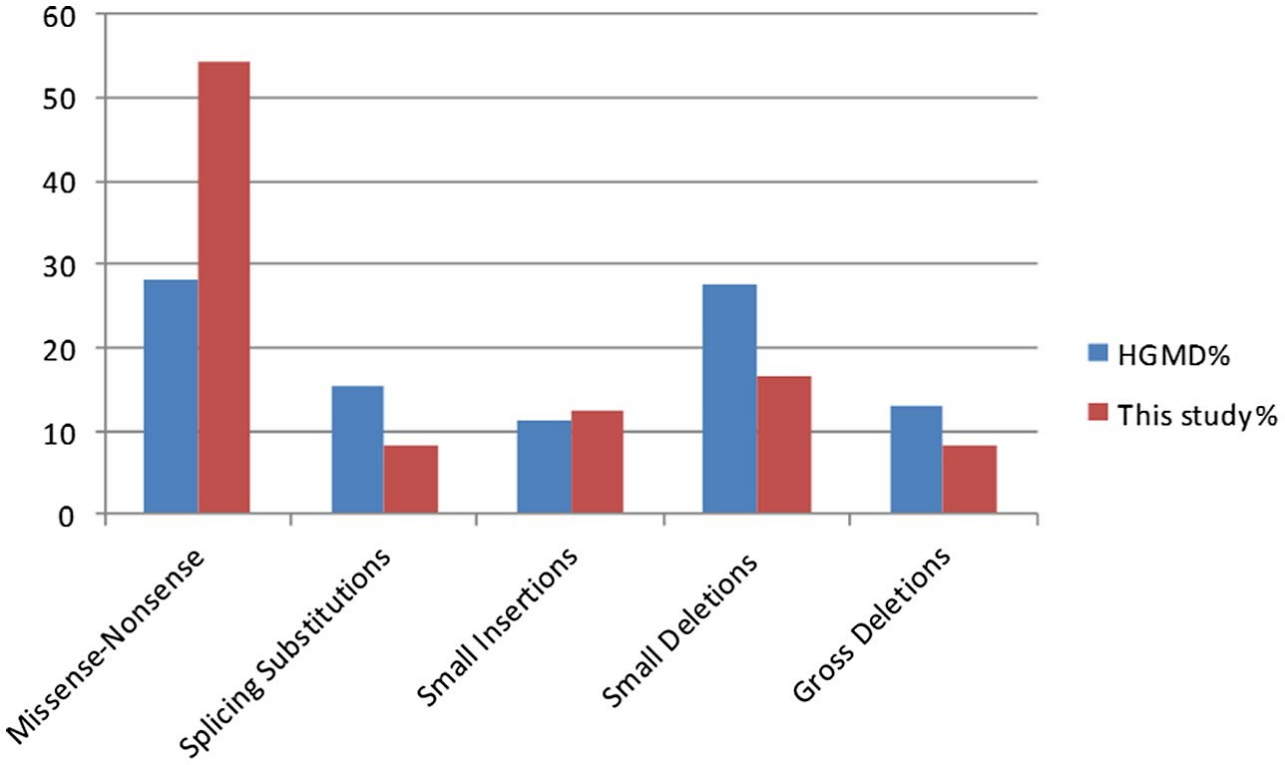
Proportion of each mutation type in this study in relation to HGMD database

**FIGURE 4:**
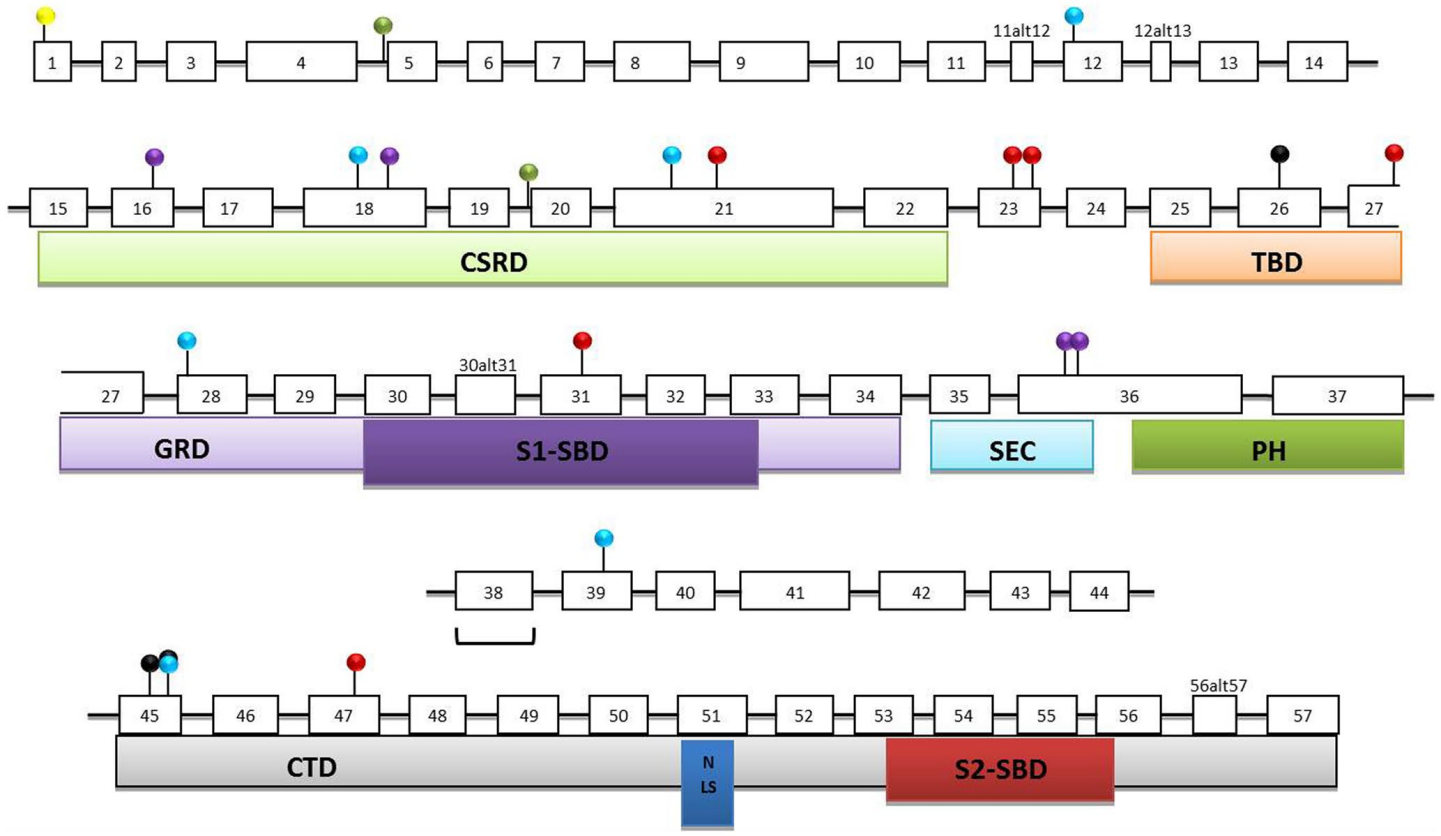
Distribution of 20 small *NF1* mutations and one genomic deletion on several *NF1* exons identified in Egyptian patients. The type of mutation is indicated using different ball color. Exon numbering is according to the new consensus system. CSRD, Cysteine Serine Rich Domain; CTD, C‐Terminal Domain; GRD, GTPase Activating Protein Related Domain; NLS, Nuclear Localization Site; PH, ‐ Pleckstrin Homology Domain; S1‐SBD, S1‐ Syndecan Binding Domain 1; S2‐SBD, S2‐ Syndecan Binding Domain; SEC, SEC14p Homology Domain; TBD, Tubulin Binding Domain. 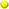, Start loss; 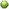, Splice site; 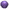, Deletion; 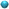, Nonsense; 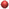, Missense; 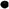, Insertion; 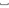, Genomic gross deletion

The frequency of each NF1 mutation type was variable among different study cohorts (Nemethova et al., 2013). In comparison to HGMD database (Human Gene Mutation Database Professional, 2019), the Egyptian studied cohort showed a significantly higher frequency of missense/nonsense mutations (54% versus 28.1%), and a lower one for small deletions (16.7% versus 27.5; Figures 3 and 4).

Finally, the causative mutation could not be detected in only one patient (4%). This might be due to the inability to detect genetic variants residing in the regulatory, the flanking intronic, or the deep intronic non‐coding regions, large genomic rearrangements or epigenetic alterations. It is foreseeable that the combination of genomic DNA and cDNA (mRNA) analyses together with testing for copy number variations could increase the detection rate of NF1 mutation detection (Evans et al., 2016).

In conclusion, the current study has presented a useful diagnostic approach for molecular analysis of *NF1* gene leading to a relatively high mutation detection rate that might help in prenatal diagnosis and control of the disease. The study has also evidenced the disease clinical variability and possible genotype‐phenotype correlations in some studied NF1 patients.

## CONFLICT OF INTEREST

The authors have declared no conflict of interest.

## AUTHORS’ CONTRIBUTION

G.Y.E., K.S.A. and M.A.E.: Study design. G.Y.E.: Clinical case selection & evaluation. N.N.A., K.S.A., R.K., M.A.E., H.R.M. and Y.Z.G.: Experimental approach design. N.N.A.: Primer design. N.N.A.: Nucleic acid extraction, cDNA Sequencing and MLPA analysis. K.S.A., R.K., H.R.M.: supervision of experimental work. K.S.A., R.K., M.A.E. and Y.Z.G.: molecular data verification. N.N.A. and R.K.: In silico analysis. G.Y.E., Y.Z.G., R.K. and K.S.A.: phenotype‐genotype correlation assessment. N.N.A.: Preparation of manuscript first draft. Y.Z.G., K.S.A., G.Y.E., R.K. and M.A.E.: Editing of manuscript versions and final revision. All authors read and approved the final manuscript.

## ETHICAL APPROVAL

This study was approved by the Medical Research Ethics Committee at the National Research Center (Reference Number: 15‐221). A written consent was obtained from all participants in accordance with the ethical standards of the institutional ethical committee and with the 1964 Helsinki declaration and its later amendments.

## Data Availability

The data that support the findings of this study are available from the corresponding author upon reasonable request.
